# Genetic Code Expanded T Cell for Controllable Immunotherapy

**DOI:** 10.1002/advs.75501

**Published:** 2026-05-04

**Authors:** Xue Wang, Yingao Gao, Yeyu Su, Yong Wang, Tao Liu

**Affiliations:** ^1^ State Key Laboratory of Natural and Biomimetic Drugs, Chemical Biology Center, Institute of Advanced Clinical Medicine, Department of Molecular and Cellular Pharmacology, School of Pharmaceutical Sciences Peking University Beijing China; ^2^ Cancer Center, Department of Medical Oncology and Radiation Sickness Peking University Third Hospital Beijing China

**Keywords:** chimeric antigen receptors (CARs), genetic code expansion (GCE), immunotherapies, noncanonical amino acids (ncAAs), T cells

## Abstract

Chimeric antigen receptor (CAR)‐T cell therapy has demonstrated curative potential against hematologic malignancies, but its clinical application remains constrained by the risk of uncontrolled immune activation. To address this, we engineered a translational control system for CAR expression based on Genetic Code Expansion (GCE), enabling tight, dose‐dependent, and function‐preserving regulation through nonsense codon suppression via noncanonical amino acids (ncAAs). By introducing amber stop codons into CAR constructs and engineered aminoacyl‐tRNA synthetase and tRNA pair, we developed a leak‐free regulatory module applicable in both Jurkat and primary human T cells. NcAA‐treated GCE‐CAR‐T cells exhibited antigen‐specific cytotoxicity and cytokine secretion comparable to wild‐type CAR‐T cells. In a xenograft mouse model, tumor‐specific immune responses were observed only upon ncAA administration, with untreated controls showing no therapeutic effect. This work establishes a stringent, fast‐acting translational switch that enables precise modulation of CAR‐T cell function without compromising efficacy, offering a promising platform for next‐generation programmable cell therapies.

## Graphical abstract

Our GCE‐CAR‐T cells enables tight, dose‐dependent, and function‐preserving control of CAR expression at the translational level through amber codon suppression and genetic incorporation of ncAA.

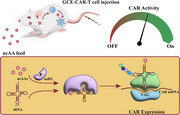

## 1 Introduction

Chimeric antigen receptor (CAR)‐T cell therapy represents a transformative advance in cancer immunotherapy, delivering durable remissions in patients with otherwise treatment‐refractory hematologic malignancies [1]. As of early 2025, six CAR‐T products (Kymriah, Yescarta, Tecartus, Breyanzi, Abecma, and Carvykti) targeting CD19 or BCMA antigens have received FDA approval for B‐cell leukemias, lymphomas, and multiple myeloma [2, 3, 4, 5, 6]. Despite these clinical milestones, the broader clinical deployment remains limited by the challenge of balancing therapeutic dosing with treatment‐related toxicities. Real‐world data have shown that over 70% of treated patients develop cytokine release syndrome (CRS)—a systemic hyperinflammatory response characterized by fever, hypotension, and multi‐organ dysfunction [7, 8, 9]. These toxicities, primarily driven by uncontrolled CAR‐T activation, underscore the urgent need for precise, tunable mechanisms to regulate therapeutic T cell function [10, 11].

To mitigate these risks, various regulatory “switch” strategies have been developed to pharmacologically modulate CAR expression or signaling [7, 12]. For instance, inducible suicide switches, such as iCaspase‐9, enable the rapid ablation of CAR‐T cells upon the administration of a chemical dimerizer and have progressed to early‐phase clinical trials [13, 14]. Synthetic Notch (synNotch) receptors implement logic‐gated control to restrict CAR activation to dual‐antigen environments. Additionally, systems like SNIP‐CARs, ligand‐inducible degrons, and split CAR architectures offer reversible and near‐binary control of CAR function in response to small‐molecule cues [15, 16, 17, 18, 19, 20]. However, despite their preclinical promise, most designs have failed to translate successfully in clinical settings. Common limitations include reduced antitumor potency, leaky background expression, slow transcriptional response kinetics, or non‐human components that raise immunogenicity concerns. Several systems have been discontinued due to dose‐limiting toxicities, underscoring the challenge of achieving stringent yet effective control [21, 22, 23, 24]. These shortcomings highlight the need for alternative regulatory modalities that can provide fast, robust, and precise control without compromising therapeutic efficacy.

Genetic code expansion (GCE) provides a fundamentally distinct regulatory strategy by enabling translational‐level control of protein synthesis [25, 26, 27]. GCE systems employ an orthogonal aminoacyl‐tRNA synthetase/tRNA pair to incorporate a specific noncanonical amino acid (ncAA) in place of an amber stop codon (TAG) [28], thereby gating protein translation in a strictly ncAA‐dependent manner [29]. This mechanism has been successfully adapted for mammalian systems, facilitating rapid, reversible, and non‐leaky regulation of diverse biological functions [30, 31]. Prior applications include biosafety containment, temporal protein control, programmable virus attenuation, and regulated therapeutic transgenes in models of diabetes and Duchenne muscular dystrophy [32, 33, 34, 35, 36, 37, 38]. Given these attributes, GCE represents a highly attractive platform for the precise control of complex cellular therapies [37, 39].

In this study, we engineered a translationally gated CAR‐T platform utilizing GCE, rendering CAR expression strictly contingent on the administration of a specific ncAA. By introducing targeted amber codon into defined extracellular or intracellular CAR domains, coupled with optimization of tRNA expression and synthetase activity, we achieved robust and tunable CAR expression with negligible background leakiness. We established stable Jurkat and primary human T cell lines using *piggyBac*‐mediated integration [40]. Functional assays confirmed that ncAA‐treated cells exhibited potent cytotoxicity and cytokine secretion comparable to conventional CAR‐T cells. In a xenograft model, tumor‐specific immune responses were observed only upon ncAA administration, confirming the system's stringency and functional integrity in vivo. Collectively, these findings establish a rapid, stringent, and function‐preserving translational switch for CAR‐T cells, providing a versatile platform for the development of next‐generation programmable immunotherapies.

## 2 Results

### 2.1 Selection and Validation of Amber Mutation Sites for NcAA‐Dependent Regulation of CAR Expression and Function

To establish a genetically encoded safety switch for CAR‐T cells via GCE, we developed a dual‐plasmid system comprising an orthogonal *Methanosarcina barkeri* pyrrolysyl‐tRNA synthetase and tRNA^Pyl^ pair (*Mb*PylRS/tRNA^Pyl^) and an amber‐mutated CAR construct [41]. A third‐generation anti‐CD19 CAR was C‐terminally fused to EGFP to enable the visualization and quantification of full‐length protein expression [42, 43]. In parallel, a Myc tag was appended to the C‐terminus of the extracellular single‐chain variable fragment (scFv) to facilitate surface detection. Amber stop codons (TAG) were systematically introduced at defined positions to enable genetic incorporation of the ncAA Nε‐(tert‐butoxycarbonyl)‐L‐lysine (BocK) (Extended Data Figure S1i) [44]. We selected BocK as the proof‐of‐concept ncAA owing to its excellent orthogonality and robust incorporation efficiency within mammalian systems, establishing a reliable and stringent baseline for evaluating the regulatory capacity of our GCE‐CAR platform.

**FIGURE 1:**
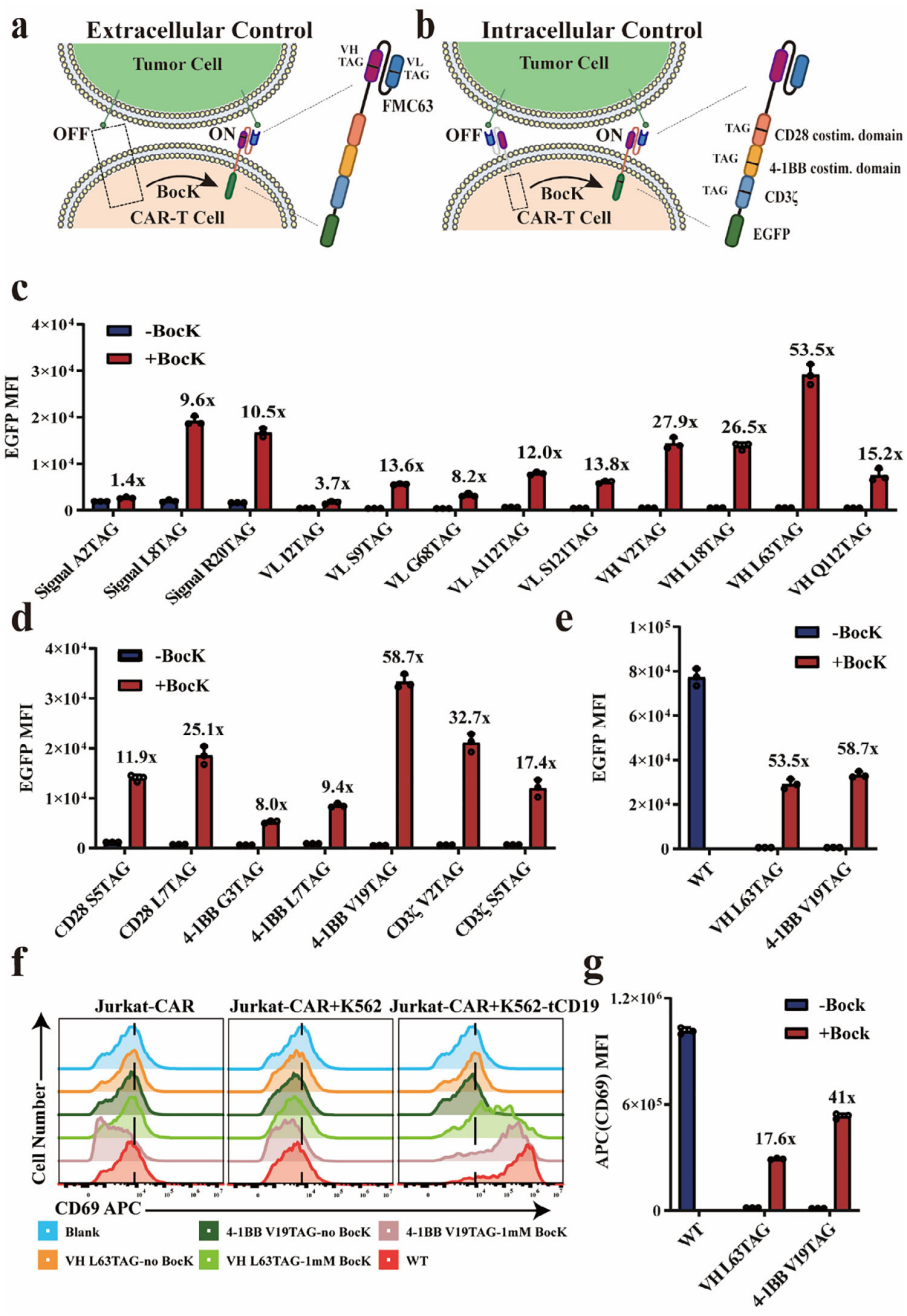
Genetic incorporation of ncAAs via an expanded genetic code enables translational control of CAR expression and activation via extracellular or intracellular regulatory mechanisms. (a, b) Schematic illustrations of GCE‐mediated control of CAR expression. Amber stop codons (TAG) were introduced into either the extracellular scFv domain (a) or the intracellular signaling domains (b) of a third‐generation anti‐CD19 CAR construct. In the absence of BocK, translation terminates at the TAG codon, preventing full‐length CAR expression. Supplementation with BocK enables site‐specific ncAA incorporation and restores functional CAR translation. (c)MFI of EGFP in JT cells transiently co‐transfected with codon‐mutated CAR constructs and a plasmid encoding *Mb*PylRS and a U6‐driven tRNA^Pyl^_CUA_. TAG codons were inserted at various extracellular sites, including the signal peptide, VL, and VH domains. CAR expression was assessed via EGFP fluorescence with or without 1 mM BocK. (d) EGFP MFI of intracellular TAG variants introduced into the CD28, 4‐1BB, or CD3ζ signaling domains. (e) Comparison of EGFP MFI between wild‐type CAR and the two top‐performing TAG variants: VH L63TAG and 4‐1BB V19TAG, which achieved 38% and 43% of wild‐type CAR expression in the presence of BocK. (f) Flow cytometry histograms depicting CD69 upregulation in CAR‐expressing JT cells after 24 h of co‐culture with CD19^−^ or CD19^+^ K562 cells, with or without 1 mM BocK. (g) Quantification of CD69 MFI from (f), confirming that VH L63TAG and 4‐1BB V19TAG variants mediate antigen‐specific, BocK‐dependent activation. Data are presented as mean ± SD from three independent biological replicates (n  =  3). Fold‐change values are annotated where applicable.

To validate the versatility of GCE‐CAR regulation, we implemented two complementary strategies based on the site‐specific incorporation of ncAAs: extracellular control and intracellular control. For extracellular control, TAG codons were introduced into the scFv domain, rendering full‐length CAR expression—and thus antigen recognition—strictly dependent on the presence of BocK (Figure 1a). In the absence of BocK, translation is prematurely terminated, resulting in CAR‐T cell inactivation. To identify optimal TAG insertion sites, we screened twelve candidate positions within the extracellular domain. Nine of these sites within the scFv region were selected based on AlphaFold‐predicted structural models of the FMC63 antibody [45] (Extended Data Figure S1a), prioritizing residues anticipated to tolerate TAG substitution without impairing folding or function. Following transient transfection into Jurkat T (JT) cells, the variants were evaluated for full‐length CAR expression via EGFP fluorescence and Myc surface staining. While all GCE variants demonstrated robust BocK‐dependent expression, three constructs with TAG insertions within the signal peptide exhibited background signal leakiness in the absence of BocK (Figure 1c; Extended Data Figure S1e). Notably, the VH L63TAG variant yielded the highest EGFP mean fluorescence intensity (MFI) alongside minimal background signal in the absence of BocK, achieving a signal‐to‐noise ratio exceeding 50‐fold (Figure 1c). Although Myc staining confirmed high surface expression for the L8TAG and R20TAG variants in the presence of BocK, EGFP analysis revealed higher background leakage for these mutants. Consequently, VH L63TAG was selected as the optimal extracellular control site due to its favorable expression profile and stringent ncAA dependency.

For the intracellular control system (Figure 1b), TAG codons were introduced within the intracellular signaling domains. In this configuration, the absence of BocK yields a truncated CAR lacking intracellular signaling capacity, which either remains anchored to the cell surface or undergoes rapid degradation due to instability. Upon BocK supplementation, translation of the full‐length CAR is restored, thereby rescuing functional signaling. Guided by AlphaFold structural predictions [45] (Extended Data Figure 1b–d), we selected seven TAG sites predicted to exert minimal impact on protein folding and function. Following transient transfection into JT cells, several variants showed strong ncAA‐dependent expression, with 4‐1BB V19TAG and CD3ζ V2TAG demonstrating the most pronounced regulation (Figure 1d). Among them, 4‐1BB V19TAG exhibited the highest MFI and signal‐to‐noise ratio (58.7‐fold), followed by CD3ζ V2TAG (32.7‐fold) and CD28 L7TAG (25.1‐fold). Immunofluorescence analysis further validated efficient BocK incorporation at these sites (Extended Data Figure S1f). Interestingly, Myc staining revealed partial surface localization for most intracellular TAG variants even in the absence of BocK, indicating that these prematurely truncated CARs remain membrane‐anchored. However, the 4‐1BB V19TAG variant emerged as a notable exception. The negligible surface Myc staining observed for this variant without BocK suggests that the resulting truncated fragment is intrinsically unstable and rapidly targeted for proteolytic degradation (Extended Data Figure S1h). Collectively, the EGFP and Myc profiling established 4‐1BB V19TAG as the optimal intracellular control site. Furthermore, its overall expression level reached 43% of the wild‐type CAR, surpassing that of the extracellular VH L63TAG variant (Figure 1e).

To evaluate the functional activity of these engineered receptors, we measured CD69 upregulation as a marker of antigen stimulation [46]. JT cells expressing either VH L63TAG or 4‐1BB V19TAG variants were co‐cultured with CD19^−^ K562 or CD19^+^ K562 cells, with or without BocK. CD69 expression was exclusively induced in the dual presence of BocK and the target antigen, confirming that CAR activation is strictly gated by ncAA availability (Figure 1f). Notably, the 4‐1BB V19TAG variant induced a stronger CD69 upregulation, achieving 53% of wild‐type activity, consistent with its higher expression level (Figure 1g). Both extracellular and intracellular strategies offer effective and tunable regulatory modalities, providing a versatile platform for genetically encoded safety switches in CAR‐T cell therapies.

### 2.2 Systematic Optimization of the GCE System for Efficient ncAA‐Dependent CAR Expression in Stable Cell Lines

**FIGURE 2:**
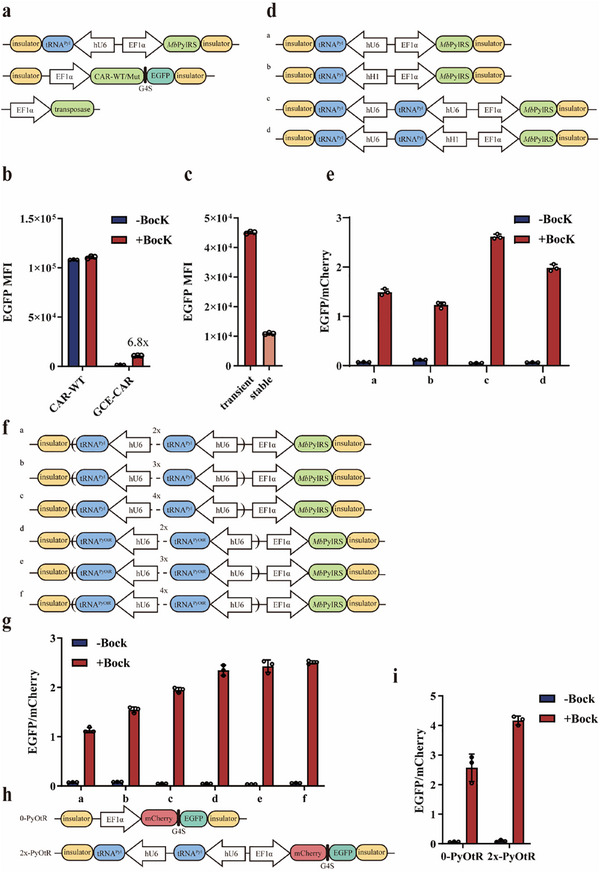
Optimization of the GCE system enhances ncAA incorporation efficiency and supports switchable CAR expression in stable mammalian cells. (a) Schematic of the *piggyBac* transposon‐based system used to generate stable GCE‐CAR‐T cells. The system comprises three plasmids: (1) a CAR‐EGFP construct carrying a TAG codon (GCE‐CAR); (2) a GCE module encoding the *Mb*PylRS and a single U6‐driven tRNA^Pyl^_CUA_; and (3) a helper plasmid expressing *piggyBac* transposase. These components were co‐electroporated for stable genomic integration. (b) EGFP MFI of JT cells stably expressing wild‐type or GCE‐CAR, cultured with or without 1 mM BocK. The resulting stable line, GCE‐CAR‐JT‐V1, exhibited a 6.8‐fold BocK‐dependent increase in EGFP signal. (c) Comparison of EGFP MFI between transiently transfected and stably integrated GCE‐CAR constructs. The stable GCE‐CAR‐JT‐V1 line exhibited substantially lower fluorescence. (d) Schematic of four GCE system variants (a–d) designed to assess the effect of Pol III promoter identity (U6 vs hH1) and tRNA^Pyl^_CUA_ copy number on suppression efficiency. All constructs co‐express *Mb*PylRS under EF1α control. (e) Quantification of ncAA incorporation using a dual‐fluorescence mCherry‐TAG‐EGFP reporter. The EGFP/mCherry ratio reflects amber suppression efficiency. Construct (c), with two U6‐driven tRNA^Pyl^_CUA_ copies, showed the highest readthrough. (f) Design of constructs (a–f) for systematic optimization of tRNA variant and gene dosage. Variants encode 2–4 copies of wild‐type tRNA^Pyl^ or the evolved PyOtR sequence, each driven by U6 promoters. (g) Reporter assay results from (f) showing EGFP/mCherry ratios. Suppression efficiency increased with tRNA copy number and plateaued beyond three copies. PyOtR consistently outperformed the wild‐type tRNA^Pyl^. (h) Schematic of a reporter plasmid incorporating two U6‐driven PyOtR copies directly into the mCherry‐TAG‐EGFP construct, boosting intracellular tRNA availability. (i) EGFP/mCherry ratios from cells transfected with reporter constructs in (h). Co‐expression of 2× PyOtR in both GCE and reporter modules significantly enhanced amber suppression. Data represent mean ± SD from three biologically independent replicates (n = 3). Fluorescence intensities were measured by flow cytometry. Fold‐change values are indicated above bars.

To evaluate the capacity of the GCE system to support stable and strictly regulated CAR expression in mammalian cells, we established a JT cell line using a three‐plasmid *piggyBac* transposon system. This system comprised: (1) an expression plasmid for the orthogonal *Mb*PylRS and its cognate tRNA^Pyl^, (2) a second plasmid encoding a TAG‐mutated CAR construct fused to EGFP (4‐1BB V19TAG), and (3) a helper plasmid expressing the hyperactive *piggyBac* transposase (Figure 2a) [40]. Following two rounds of fluorescence‐activated cell sorting (FACS), the resulting GCE‐CAR‐JT‐V1 stable cell line exhibited robust BocK‐dependent CAR expression with minimal background leakage in the absence of ncAA (Figure 2b). However, total CAR expression reached only ≈10% of wild‐type levels and approximately 24% of that observed in transiently transfected cells (Figure 2b, c). This drop highlights a critical translational bottleneck in ncAA incorporation efficiency upon stable genomic integration.

Given the critical importance of sufficient CAR expression for T cell activation and therapeutic efficacy, we systematically optimized the GCE system to improve amber suppression efficiency. Previous reports have indicated that robust suppression in mammalian systems may require integration of over 400 tRNA gene copies [47]. Accordingly, we explored multiple optimization strategies including increasing tRNA gene dosage, evaluating alternative RNA polymerase III promoters [48], and utilizing an engineered tRNA variants [49]. To precisely quantify these improvements, we employed a dual‐fluorescence mCherry‐TAG‐EGFP reporter construct, wherein an amber stop codon separates the two fluorophores. In this system, mCherry serves as an internal control for transfection efficiency, while EGFP fluorescence directly reflects successful ncAA incorporation. The EGFP/mCherry fluorescence ratio thus provides a sensitive readout of incorporation efficiency.

Initial optimizations focused on tRNA promoter selection and gene dosage (Figure 2d). Comparative analysis revealed that two copies of U6‐driven tRNA^Pyl^ yielded the highest EGFP/mCherry ratios. This confirmed both the superiority of the U6 promoter and the functional benefit of increasing tRNA copy number (Figure 2e; Extended Data Figure S2a). We next evaluated PyOtR, an evolved tRNA variant previously engineered through directed evolution for enhanced amber suppression (Figure 2f). While increasing the PyOtR copy number from two to four further enhanced readthrough, the improvement plateaued beyond three copies. The configuration containing two U6‐driven PyOtR units provided strong suppression with minimal plasmid burden, rendering it optimal for downstream applications (Figure 2g; Extended Data Figure S2b).

To further boost tRNA availability, we integrated two additional copies of U6‐driven PyOtR into the reporter plasmid itself (Figure 2h). This configuration, designated the “2×‐PyOtR” system, significantly enhanced EGFP signal compared to earlier constructs (Figure 2i; Extended Data Figure S2c), demonstrating that increasing tRNA abundance within both the orthogonal synthetase machinery and the reporter module synergistically improves ncAA incorporation efficiency. Through these iterative optimizations, we established a robust, streamlined GCE platform capable of driving consistent, high‐level, and strictly ncAA‐dependent protein expression in stably transduced mammalian cells. We adopted this optimized system in all subsequent experiments for engineering tightly regulated CAR‐T cell platforms.

### 2.3 Precise Regulation of CAR Expression and Activation in Stable Jurkat T Cells

**FIGURE 3:**
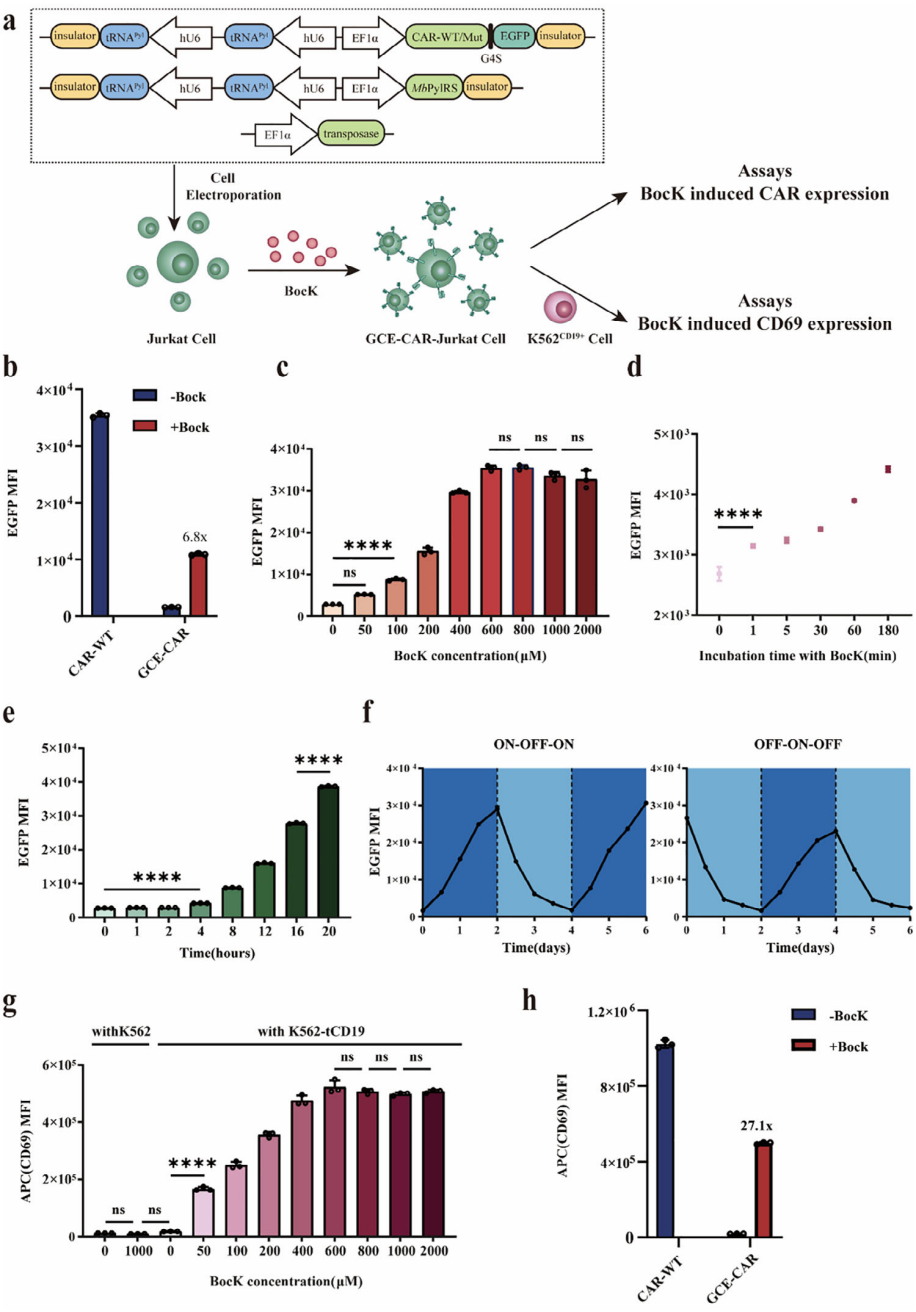
The optimized GCE system enables rapid, reversible, and dose‐dependent translational control of CAR expression and antigen‐specific activation in stable Jurkat T cells. (a) Schematic representation of the *piggyBac*‐based genome integration strategy used to generate stable GCE‐CAR‐Jurkat cells. Three plasmids were co‐electroporated: (1) a third‐generation anti‐CD19 CAR construct carrying an amber codon at position 19 of the 4‐1BB domain and fused to EGFP; (2) a GCE module encoding *Mb*PylRS and two U6‐driven copies of the evolved tRNA^Pyl^ variant PyOtR; and (3) a *piggyBac* transposase helper plasmid. Clonal lines were isolated using dual fluorescence‐activated cell sorting (FACS). (b) Flow cytometry analysis of EGFP MFI in stable JT cells expressing either wild‐type or TAG‐mutated CAR, with or without 1 mM BocK. The engineered line GCE‐CAR‐JT‐V2 exhibited a 6.8‐fold increase in EGFP signal upon BocK addition, accounting for approximately 30% of wild‐type expression. (c) Dose‐dependent activation of CAR expression in GCE‐CAR‐JT‐V2 cells exposed to increasing concentrations of BocK (0–2000 µM) for 48 h. A measurable induction of EGFP fluorescence was detected as low as 100 µM, with saturation observed above 600 µM, indicating robust translational responsiveness to ncAA concentration. (d) Transient exposure assay assessing system sensitivity. Cells were pulsed with 1 mM BocK for 1 to 180 minutes, followed by 8 h incubation in BocK‐free medium. Even 1‐minute exposure was sufficient to trigger detectable CAR expression, underscoring the system's responsiveness to brief ncAA availability. (e) Time‐course analysis of CAR induction following continuous 1 mM BocK treatment. EGFP signal became detectable within 4 h and steadily increased over 20 h, demonstrating rapid protein production kinetics under translational control. (f) Reversible regulation of CAR expression using alternating BocK administration. Under both ON–OFF–ON and OFF–ON–OFF regimens, EGFP expression dynamically tracked ncAA availability, with fluorescence levels returning to baseline within 48 h of BocK withdrawal. (g) BocK dose‐dependent JT activation in response to CD19^+^ K562 target cells. After 48 h of co‐culture, CD69 surface expression was upregulated only in cells treated with BocK and co‐incubated with CD19‐expressing targets, confirming both antigen‐ and ncAA‐dependent CAR function. (h) Quantification of CD69 MFI in CAR‐WT and GCE‐CAR‐JT‐V2 cells following co‐culture with CD19^+^ K562 cells. BocK‐induced GCE‐CAR‐JT‐V2 cells reached 49% of wild‐type activation levels and exhibited a 27.1‐fold increase over untreated controls. Data are presented as mean ± SD (n = 3 biologically independent replicates). Statistical significance was assessed using one‐way ANOVA. Significance levels are indicated as follows: *P < 0.05, **P < 0.01, ***P < 0.001, **** *P* < 0.0001; ns, not significant. Fold‐change values are annotated above the corresponding bars.

To functionally validate the optimized GCE system, we established a second‐generation stable JT cell line, designated GCE‐CAR‐JT‐V2, by integrating the dual‐vector *Mb*PylRS/2×U6‐PyOtR machinery alongside the target CAR construct via the *piggyBac* transposon system (Figure 3a). In the presence of 1 mM BocK, this cell line exhibited robust, ncAA‐dependent CAR expression, reaching approximately 30% of wild‐type levels based on EGFP fluorescence (Figure 3b). This represents a substantial improvement over the first‐generation construct (which achieved only ≈10%), confirming that our systematic optimization successfully mitigated the translational bottleneck and enhanced amber suppression efficiency.

We next systematically characterized the regulatory properties of the GCE‐CAR‐JT‐V2 line, focusing on responsiveness, reversibility, and sensitivity. Titration experiments revealed clear dose‐dependent induction of CAR expression, with saturation observed above 600 µM BocK (Figure 3c; Extended Data Figure S3a). Remarkably, a mere 1‐minute pulse exposure to BocK followed by an 8‐hour incubation in ncAA‐free medium was sufficient to induce detectable EGFP expression (Figure 3d; Extended Data Figure S3b), highlighting the system's exceptional sensitivity to transient ncAA availability. Furthermore, time‐course analysis demonstrated that CAR expression became detectable within 4 hours of BocK addition and increased steadily over time (Figure 3e; Extended Data Figure S3c). To assess the reversibility of the translational switch, we subjected the cells to alternating ON‐OFF‐ON and OFF‐ON‐OFF cycles by modulating BocK availability in the culture medium. EGFP expression closely followed BocK availability, exhibiting rapid induction upon addition and decay to baseline within 48 hours of withdrawal (Figure 3f; Extended Data Figure S3d). These results demonstrate the system's capacity for tight, temporally resolved regulation of CAR expression.

Subsequently, we further investigated functional consequences by co‐culturing GCE‐CAR‐JT‐V2 cells with CD19^+^ K562 cells across a BocK concentration gradient. Consistent with the expression data, CD69 expression was induced in a strictly BocK dose‐dependent manner (Figure 3g; Extended Data Figure S3e). No observable activation occurred in the absence of BocK or when co‐cultured with CD19^−^ K562 cells. Crucially, compared to the uninduced state, BocK administration triggered a striking 27.1‐fold enhancement in CD69 surface expression (Figure 3h). This confirms that both ncAA incorporation and antigen engagement are required for CAR‐mediated activation, effectively functioning as a high‐fidelity “AND‐gate” safety switch.

### 2.4 NcAA‐Dependent Regulation of CAR Expression and Function in Primary Human T Cells

**FIGURE 4:**
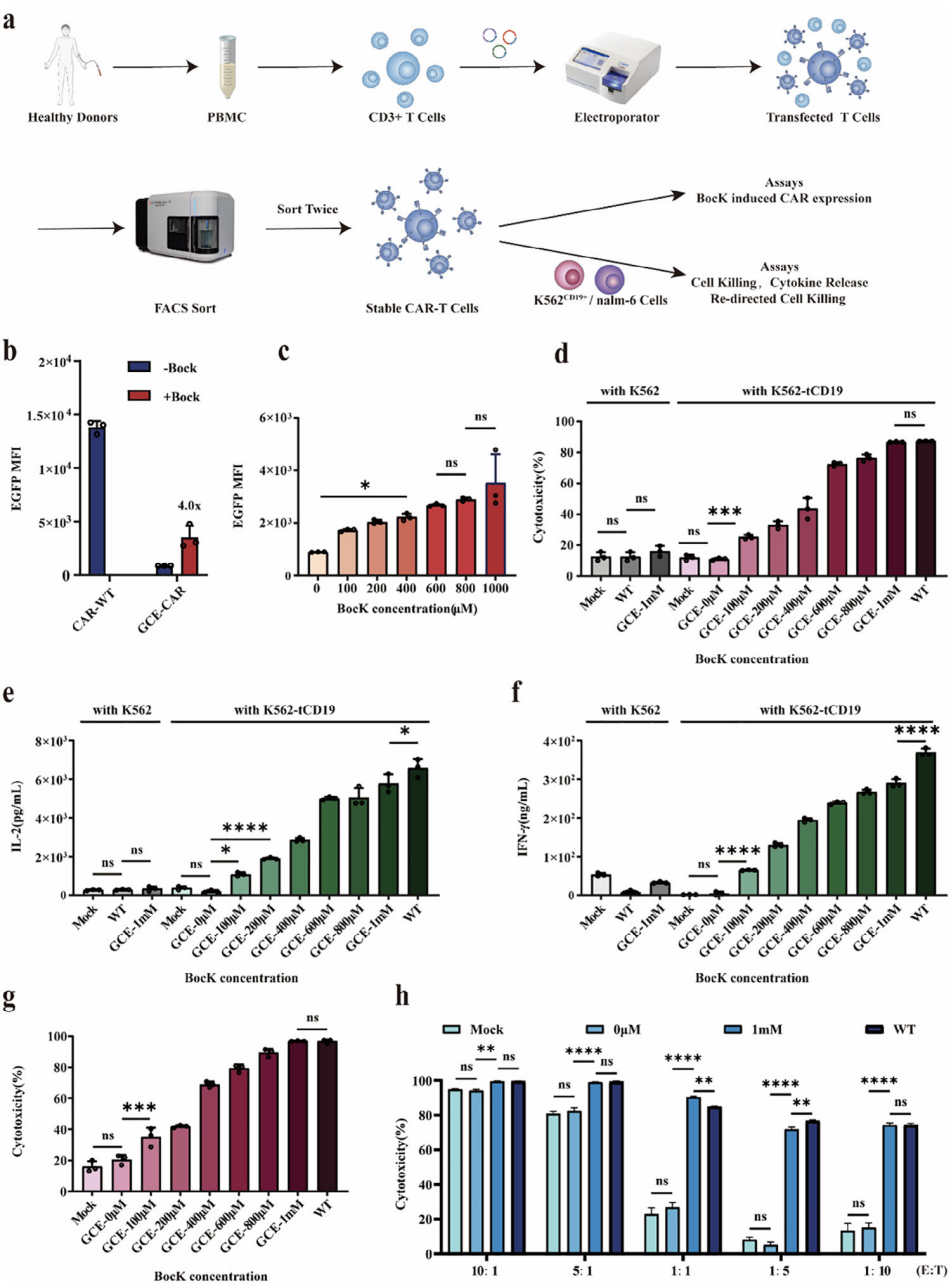
NcAA‐dependent regulation of CAR expression and cytotoxicity function in primary human T cells. (a) Schematic workflow for engineering GCE‐CAR‐T cells from healthy donor‐derived CD3^+^ T cells. Activated T cells were electroporated with three *piggyBac*‐based plasmids encoding (1) a 4‐1BB‐V19TAG‐mutated anti‐CD19 CAR fused to EGFP and two U6‐driven PyOtR, (2) an *Mb*PylRS and two U6‐driven PyOtR expression cassette, and (3) a *piggyBac* transposase helper plasmid. After two rounds of FACS enrichment, stable CAR‐T cells (termed GCE‐CAR‐T) were expanded for further evaluation. (b) EGFP MFI of wild‐type and GCE‐CAR‐T cells in the presence or absence of 1 mM BocK. BocK supplementation induced a 4.0‐fold increase in GCE‐CAR‐T fluorescence, reaching ≈25% of wild‐type expression. (c) Dose‐dependent CAR expression in GCE‐CAR‐T cells exposed to increasing BocK concentrations (0–1000 µM) for 48 h. CAR‐EGFP signal increased with BocK dose and became clearly detectable as low as 100 µM. (d) BocK‐dependent cytotoxicity of GCE‐CAR‐T cells against CD19^+^ K562 cells. At 1 mM BocK, GCE‐CAR‐T cells exhibited killing efficacy equivalent to wild‐type CAR‐T cells. No cytotoxicity was observed against CD19^−^ K562 targets or in the absence of BocK. (e, f) BocK dose‐dependent IL‐2 (e) and IFN‐γ (f) secretion by GCE‐CAR‐T cells following co‐culture with CD19^+^ K562 cells. Cytokine release at 1 mM BocK matched wild‐type levels, confirming robust antigen‐specific activation. (g) Cytotoxicity of GCE‐CAR‐T cells against the native CD19^+^ leukemia cell line Nalm‐6 across a BocK gradient. At 1 mM BocK, GCE‐CAR‐T cells achieved wild‐type killing efficiency; in the absence of BocK, killing was indistinguishable from that of the mock control group. (h) GCE‐CAR‐T cytotoxicity against Nalm‐6 targets at varying effector‐to‐target (E:T) ratios. Strong killing was observed only in the presence of BocK, and matched wild‐type levels across multiple E:T conditions. Data are presented as mean ± SD (n  =  3 independent biological replicates). Statistical significance was determined by one‐way ANOVA. Significance indicators: *P < 0.05; **P < 0.01; ***P < 0.001; ****P < 0.0001; ns, not significant.

To evaluate the GCE system in primary immune cells, we introduced the *Mb*PylRS/2×U6‐PyOtR cassette and the 4‐1BB V19 TAG‐mutated CAR construct into activated CD3^+^ T cells from healthy human donors using a *piggyBac* transposon system (Figure 4a). Following two rounds of FACS enrichment, we successfully established a stable CAR‐T cell line, designated GCE‐CAR‐T. Upon BocK supplementation, GCE‐CAR‐T cells expressed detectable levels of CAR protein, with EGFP fluorescence reaching approximately 25% of that observed in wild‐type CAR‐T cells (Figure 4b, c). Furthermore, CAR expression exhibited a clear BocK concentration‐dependent pattern (Figure 4c; Extended Data Figure S4a, b), indicating precise and tunable translational control in primary human T cells.

We next assessed the functional consequences of ncAA‐regulated CAR expression using two CD19^+^ tumor cell models. In co‐culture assays with engineered CD19^+^ K562 cells, GCE‐CAR‐T cells exhibited robust BocK dose‐dependent cytotoxicity, while no target cell killing was observed in the absence of BocK or when co‐cultured with CD19^−^ K562 controls (Figure 4d) [50]. These results confirm both the antigen specificity and ncAA dependency of the effector function. Despite the lower steady‐state receptor density, GCE‐CAR‐T cells mounted a robust and highly specific cytotoxic response against CD19^+^ K562 targets, accompanied by a dose‐dependent secretion of IL‐2 and IFN‐γ that closely approached WT levels at saturating BocK concentrations (Figure 4e, f) [51]. To validate these findings in a more clinically relevant setting, we extended our analysis to Nalm‐6, a human B‐ALL cell line that naturally expresses CD19. Consistent with previous observations, GCE‐CAR‐T cells showed a strong BocK‐dependent cytotoxic response against Nalm‐6 targets, achieving wild‐type levels of cytotoxicity at 1 mM BocK (Figure 4g; Extended Data Figure S4c). This effect was further confirmed across a range of effector‐to‐target (E:T) ratios, where potent cytotoxicity was observed exclusively under BocK supplementation, while no significant activity was detected in its absence or in mock‐treated controls (Figure 4h). To validate the reversibility and temporal control of this system, we monitored the decay of cytotoxic activity following BocK withdrawal. Upon removal of the ncAA from the culture medium, the specific killing against Nalm‐6 targets rapidly diminished to baseline levels within 48 hours (Extended Data Figure S5a, b). These results underscore the tight translational control and potent, antigen‐specific functionality of GCE‐CAR‐T cells across multiple tumor models.

To rigorously evaluate long‐term persistence and functional fitness, we subjected the engineered cells to a serial tumor re‐challenge assay [52]. Both WT and GCE‐CAR‐T cells exhibited comparable, robust cumulative expansion across three rounds of antigen stimulation, with the GCE cohort maintaining stable CAR surface expression (45%–50%) (Extended Data Figure S6a, b). Phenotypic analysis revealed that GCE‐CAR‐T cells retained a significantly higher proportion of the CD45RA^+^CD62L^+^ phenotype (typically identifying Naive, T_N_ or Stem Cell Memory T cells, T_SCM_) compared to the WT group (Extended Data Figure S6c). Together, these findings directly demonstrate that the GCE system sustains long‐term T cell fitness.

### 2.5 In Vivo Validation of NcAA‐Regulated CAR‐T Therapy in a Leukemia Xenograft Model

**FIGURE 5:**
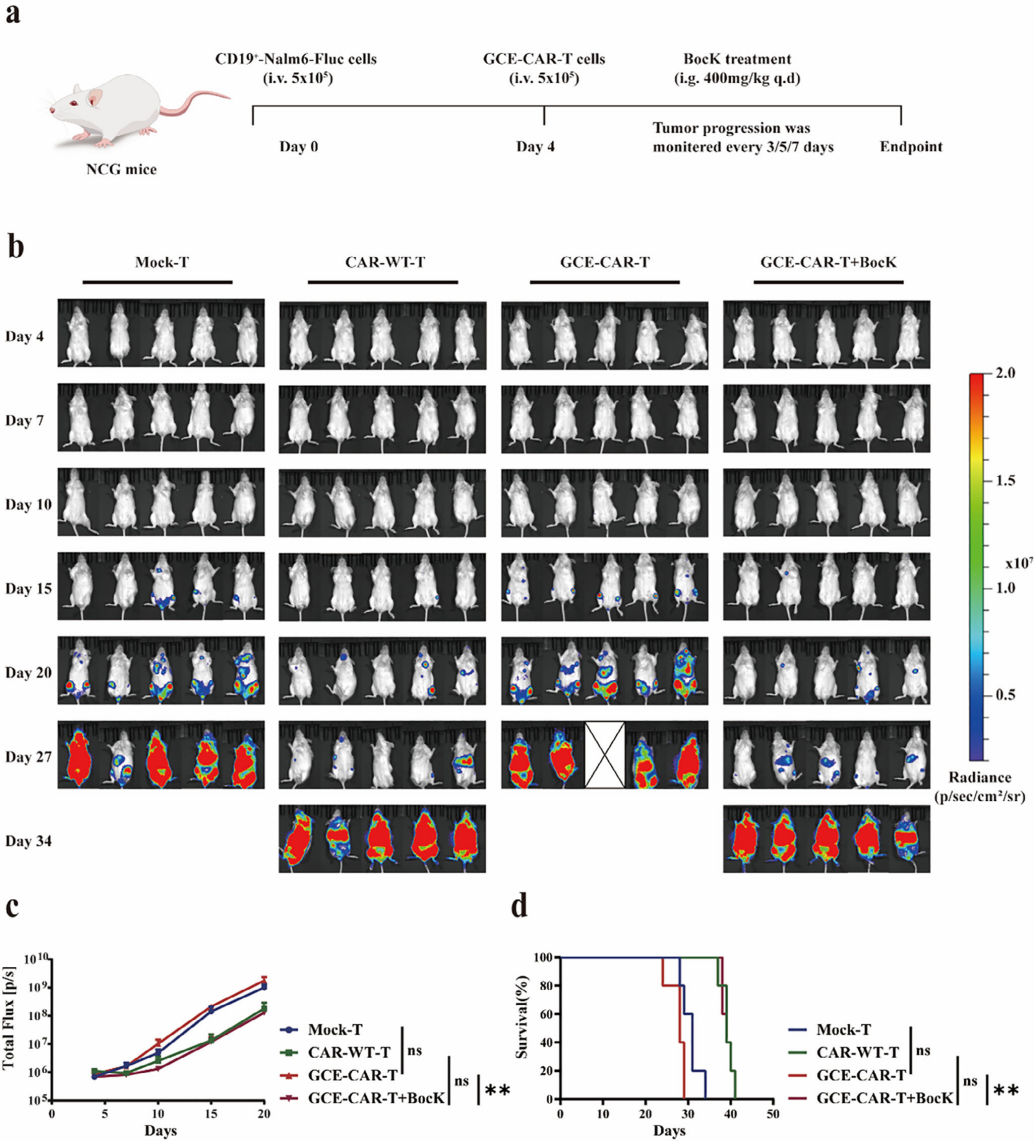
In vivo antitumor efficacy of GCE‐CAR‐T cells in a disseminated leukemia model. (a) Schematic of the in vivo experimental design. NCG mice were intravenously inoculated with 5 × 10^5^ CD19^+^ Nalm‐6‐Fluc leukemia cells on day 0. On day 4, mice were intravenously injected with 5 × 10^5^ CAR‐WT‐T cells, GCE‐CAR‐T cells, or mock T cells. Starting on the same day, mice in the indicated groups were administered BocK daily via oral gavage (400 mg/kg). Tumor progression was monitored by bioluminescence imaging every 3–7 days. (b) Representative bioluminescence images illustrating tumor burden in each group from day 4 to day 34. Treatment with GCE‐CAR‐T cells plus BocK induced robust tumor‐specific immune responses, with efficacy comparable to that of CAR‐WT‐T cells. In contrast, GCE‐CAR‐T cells without BocK showed progressive disease, similar to the control group. (c) Quantification of total bioluminescence flux (photons/sec) from whole‐body regions of interest (ROIs), measured using Living Image software. Mice receiving GCE‐CAR‐T plus BocK exhibited tumor control kinetics similar to CAR‐WT‐T cells. Data represent mean ± s.e.m. (n  =  5 mice per group). Statistical comparisons were performed using two‐way ANOVA. (d) Kaplan–Meier survival curves for each treatment group. GCE‐CAR‐T plus BocK significantly prolonged survival relative to GCE‐CAR‐T without BocK and mock groups, achieving outcomes comparable to the CAR‐WT‐T group. Statistical significance was assessed using the log‐rank (Mantel–Cox) test. *P < 0.05, **P < 0.01, ***P < 0.001, ****P < 0.0001; ns, not significant.

To evaluate the in vivo therapeutic efficacy and regulatory stringency of the GCE‐CAR‐T system, we established a disseminated leukemia xenograft model by intravenously engrafting NCG mice with CD19^+^ Nalm‐6‐Fluc cells. Four days after tumor inoculation, mice were treated with CAR‐WT‐T cells, GCE‐CAR‐T cells, or mock T cells, followed by daily oral administration of BocK (400 mg/kg) or vehicle control (Figure 5a). Tumor progression was longitudinally monitored by bioluminescence imaging.

As anticipated, CAR‐WT‐T treatment mediated robust tumor clearance, demonstrating potent baseline antitumor efficacy. Crucially, mice treated with GCE‐CAR‐T cells in combination with BocK exhibited tumor clearance kinetics and total bioluminescence flux suppression perfectly comparable to the CAR‐WT‐T cohort, confirming that ncAA supplementation does not compromise the ultimate therapeutic potency of the engineered T cells in vivo. In contrast, mice receiving GCE‐CAR‐T cells without BocK experienced unabated disease progression, yielding a tumor burden indistinguishable from that of mock‐treated controls (Figure 5b, c). This stark in vivo divergence highlights that CAR expression and its subsequent antitumor activity remain stringently gated by ncAA availability, with no biologically significant background leakiness.

Kaplan–Meier survival analysis further corroborated these imaging outcomes. Treatment with GCE‐CAR‐T cells plus BocK significantly prolonged survival relative to both the uninduced GCE‐CAR‐T and mock‐treated groups, achieving an overall survival trajectory equivalent to the WT‐CAR‐T positive control (Figure 5d). To further evaluate the systemic persistence and functional state of the engineered cells, we characterized the in vivo expansion and exhaustion profiles of the infused T cells. Flow cytometric analysis revealed no significant differences in either T‐cell proliferation or the expression of key exhaustion markers between the BocK‐induced GCE‐CAR‐T and CAR‐WT‐T cohorts (Extended Data Figure S7b, c). Collectively, these results establish that our GCE‐CAR‐T platform enables precise, binary control over CAR‐T cell activation and systemic tumor eradication in vivo, laying a robust foundation for next‐generation programmable immunotherapies.

## 3 Discussion and Conclusion

While chimeric antigen receptor (CAR)‐T cell therapy has transformed the treatment landscape for hematologic malignancies, its broader clinical application remains severely constrained by dose‐limiting toxicities and immune‐related adverse events. Although diverse regulatable architectures—including suicide switches, split CARs, and synthetic logic gates—have been developed to mitigate these risks, many fail to deliver stringent control under physiological conditions without compromising anti‐tumor efficacy. These persistent translational hurdles underscore the urgent need for alternative modalities that enable the tunable regulation of CAR‐T cell activity.

In this study, we developed a genetically encoded safety switch for CAR‐T cells utilizing genetic code expansion (GCE). By strategically introducing amber codons into the CAR construct, we achieved translational‐level gating of CAR expression strictly dependent on the administration of the noncanonical amino acid (ncAA), BocK. Through systematic optimization of tRNA architecture and the copy number of orthogonal components, our system demonstrated robust amber suppression with negligible background expression. In both stable Jurkat T cells and primary human T cells, this platform enabled rapid, stringent and highly dose‐dependent CAR expression. Crucially, although surface CAR density reached only ≈25% of wild‐type levels, GCE‐CAR‐T cells exhibited in vitro cytotoxicity and cytokine secretion comparable to wild‐type cells upon BocK induction. This capacity to maintain full therapeutic potency under tightly gated expression represents a critical advancement over existing regulatory systems, which frequently sacrifice CAR‐T cell function for controllability. Furthermore, in a Nalm‐6 leukemia xenograft model, GCE‐CAR‐T cells effectively cleared tumors in a strictly BocK‐dependent manner, achieving outcomes indistinguishable from wild‐type CAR‐T therapy, thereby validating the in vivo stringency and efficacy of the GCE translational switch.

The GCE‐based regulatory framework introduced here provides a highly modular and versatile platform for translational control of engineered immune cells. While this study establishes a robust proof‐of‐concept, the current GCE‐CAR‐T system can be further optimized to maximize its translational success. First, while robust suppression was achieved, the tRNA processing, synthetase efficiency, and cellular ncAA uptake mechanisms can be further optimized to improve incorporation efficiency and lower the required dose. Second, transitioning from the current transposon‐based delivery to more efficient, clinical‐grade vector systems (e.g., lentiviral vectors or targeted lipid nanoparticles) will be essential for maximizing therapeutic yield. Finally, future iterations of the platform could explore a wider range of ncAAs that are engineered to have customized pharmacokinetic characteristics and specific response capabilities. Integration with complementary synthetic biology tools—such as gene editing, inducible promoters, and biosensing circuits—could further amplify the responsiveness and clinical potential of these programmable cell products. With continued development, GCE‐mediated control systems may offer a compelling path toward safer, next‐generation immunotherapies.

## Author Contributions

T.L., and X.W. conceived the project. T.L. supervised the study. X.W. and Y.W. developed the stringent, site‐specific, ncAA‐dependent, translationally gated CAR‐T platform. X.W. and Y.Y.S. contributed to the Systematic Improvement of the GCE System. X.W. and Y.A.G. contributed to the in vitro and in vivo validation. X.W., Y.A.G., Y.W., and T.L. wrote the paper. All authors approved the final manuscript.

## Ethics

All experiments involving animals were conducted in accordance with the guidelines of the Peking University Animal Care and Use Committee and East China Normal University (ECNU) Animal Care and Use Committee and in direct accordance with the Ministry of Science and Technology of the People's Republic of China on Animal Care. The protocols were approved by the Peking University Animal Care and Use Committee (protocol ID DLASBD0623) and the ECNU Animal Care and Use Committee (protocol ID m20250103). All mice were euthanized upon the termination of the experiments.

## Conflicts of Interest

The authors declare no conflict of interest.

## Supporting information




**Supporting File 1**: advs75501‐sup‐0001‐SuppMat.docx.

**Supporting File 2**: advs75501‐sup‐0002‐Data.zip.

## Data Availability

The data that support the findings of this study are available from the corresponding author upon reasonable request.
