# Supplementary material for: Genetic Code Expanded T Cell for Controllable Immunotherapy
Source: Adv Sci (Weinh). 2026 May 4;13(41):e75501. doi: 10.1002/advs.75501 (PMC13335618; doi:10.1002/advs.75501)
Supplement: Supplementary file 1 — Supporting File 1: advs75501‐sup‐0001‐SuppMat.docx. [file ADVS-13-e75501-s001.docx]

Supplementary Materials for

**Genetic Code Expanded T Cell for Controllable Immunotherapy**

**Xue Wang^1,3^,** **Yingao Gao^1,3^, Yeyu Su^1^, Yong Wang^1,*^, Tao Liu^1,2，*^**

^1^State Key Laboratory of Natural and Biomimetic Drugs, Chemical Biology Center, Institute of Advanced Clinical Medicine, Department of Molecular and Cellular Pharmacology, School of Pharmaceutical Sciences, Peking University, Beijing, China

^2^Cancer Center, Department of Medical Oncology and Radiation Sickness, Peking University Third Hospital, Beijing, China

^3^These authors contributed equally: Xue Wang, Yingao Gao

*e-mail: [taoliupku@pku.edu.cn](mailto:taoliupku@pku.edu.cn); yong_wang@pku.edu.cn

**Table of contents:**

**Extended Data Figures S1-S8**

**Methods**

**SM References**

**Other supporting materials for this manuscript include the following:**

**Supplementary Table S1**

**
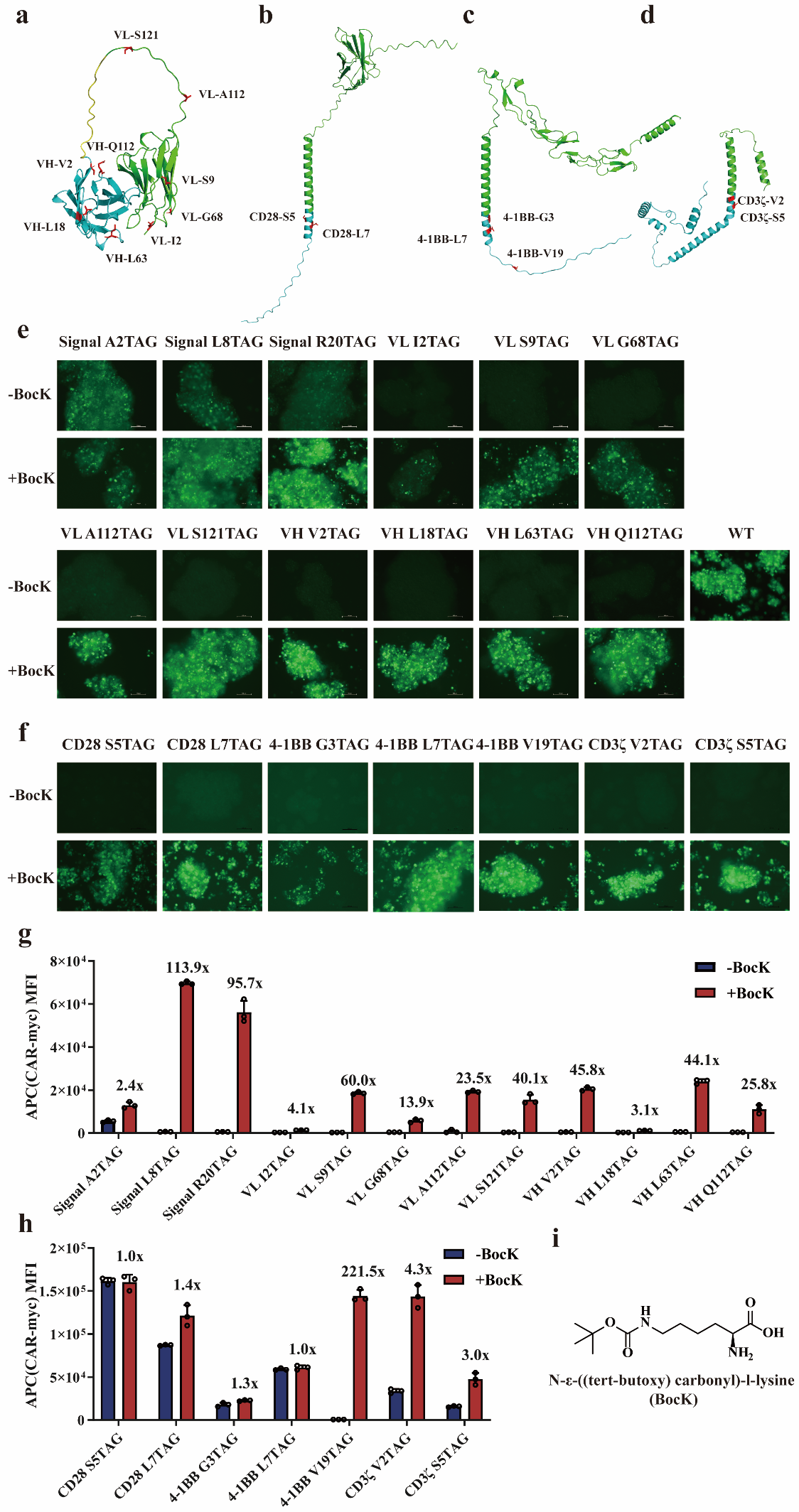
**

**Extended Data Figure S1. Structural modeling and experimental validation of TAG mutation sites for GCE-mediated regulation of CAR expression.**

(**a–d**) AlphaFold3-predicted structural models of the FMC63 scFv (**a**), and the intracellular domains of CD28 (**b**), 4-1BB (**c**), and CD3ζ (**d**). Candidate amber codon (TAG) insertion sites are highlighted in red. Positions were selected based on predicted surface accessibility and minimal disruption to local folding or functional motifs. (**e, f**) Fluorescence microscopy images of JT cells transiently co-transfected with codon-mutated CAR constructs and a plasmid encoding wild-type Methanosarcina barkeri pyrrolysyl-tRNA synthetase (MbPylRS) and one U6-driven tRNA^Pyl^_CUA_. Cells were cultured for 24 hours in the presence or absence of 1 mM BocK. Representative EGFP fluorescence images are shown for extracellular TAG variants (**e**) and intracellular TAG variants (**f**). Strong fluorescence was observed only in the presence of BocK, indicating site-specific ncAA-dependent CAR expression. WT: wild-type CAR without TAG mutation. (g, h) Quantification of Myc surface staining intensity (mean fluorescence intensity, MFI) for CAR variants with extracellular (**g**) or intracellular (**h**) TAG insertions, as detected by flow cytometry 48 hours post-transfection. JT cells were stained with anti-Myc-APC to assess surface expression of full-length CAR protein. Data are presented as mean ± SD from three biologically independent replicates (n=3). Fold-change between +BocK and –BocK conditions is indicated above each bar. (**i**) Chemical structure of the noncanonical amino acid Nε-(tert-butoxycarbonyl)-L-lysine (BocK), used for translational control of CAR expression via genetic code expansion in this study.


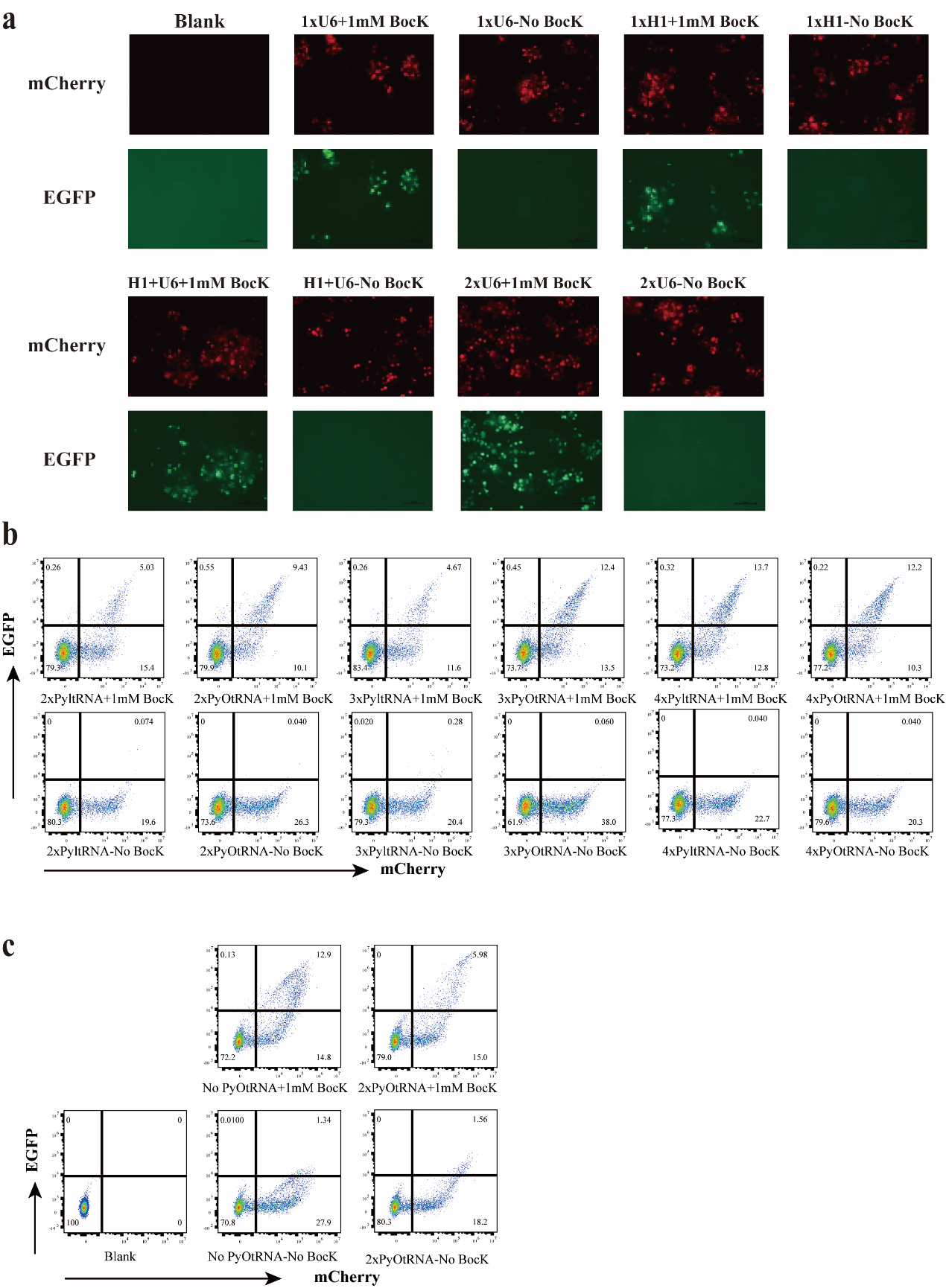


**Extended Data Figure S2. Optimization of the GCE system enhances ncAA incorporation efficiency and supports switchable CAR expression in stable mammalian cells.** (**a**) Fluorescence microscopy images of the four GCE system variants using a dual-fluorescence mCherry-TAG-EGFP reporter. The construct with two U6-driven tRNA^Pyl^_CUA_ copies exhibited the highest fluorescence intensity, suggesting that it achieved efficient readthrough. (**b**) Flow cytometry results of systematic optimization of gene dosage. (**c**) Flow cytometry results of systematic optimization of tRNA variant.


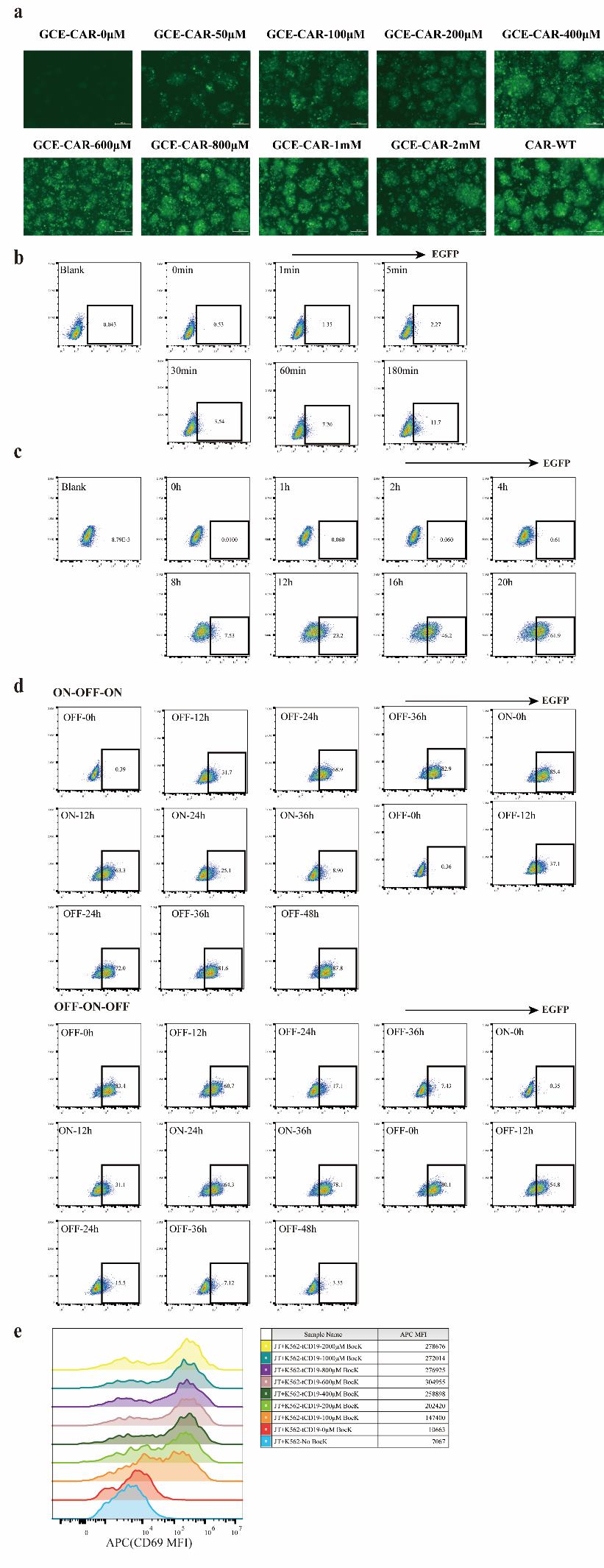


**Extended Data Figure S3. The optimized GCE system enables rapid, reversible, and dose-dependent translational control of CAR expression and antigen-specific activation in stable Jurkat T cells.** (**a**) Fluorescence microscopy images of dose-dependent CAR expression in wild-type CAR-WT-JT cells and GCE-CAR-JT-V2 cells exposed to increasing BocK concentrations (0–2000 μM) for 48 h. (**b**) Flow cytometry results of transient exposure assay from 1 to 180 minutes.(**c**) Flow cytometry results of time-course analysis of CAR induction following continuous 1 mM BocK treatment from 0h to 20h. (**d**) Flow cytometry results of reversible regulation of CAR expression using alternating BocK administration. In both ON–OFF–ON and OFF–ON–OFF regimens, EGFP expression dynamically tracked ncAA presence, with fluorescence levels returning to baseline within 48 h of BocK withdrawal. (**e**) CD69 MFI showing the activation levels of GCE-CAR-JT-V2 cells with increasing concentrations of BocK (0–2000 μM) following co-culture with K562 cells for 48h.


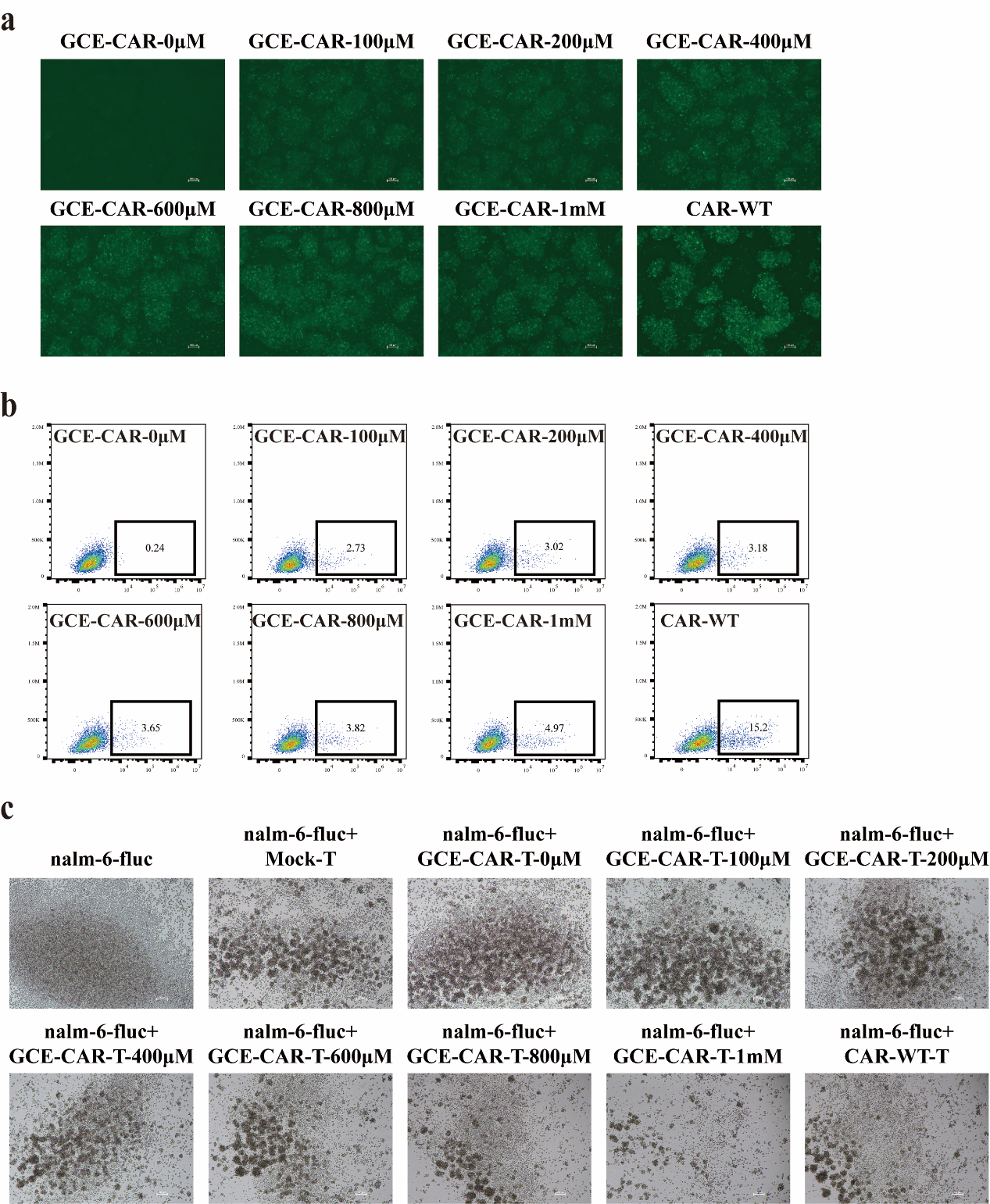


**Extended Data Figure S4. ncAA-dependent regulation of CAR expression and cytotoxicity function in primary human T cells.**

(**a**) Fluorescence microscopy images of dose-dependent CAR expression in wild-type CAR-T cells and GCE-CAR-T cells exposed to increasing BocK concentrations (0–1000 μM) for 48 h. (**b**) Dose-dependent CAR expression in wild-type CAR-T cells and GCE-CAR-T cells exposed to increasing BocK concentrations (0–1000 μM) for 48 h. (**c**) Images showing the cytotoxicity of GCE-CAR-T cells against the native CD19⁺ leukemia cell line Nalm-6 across a BocK gradient.


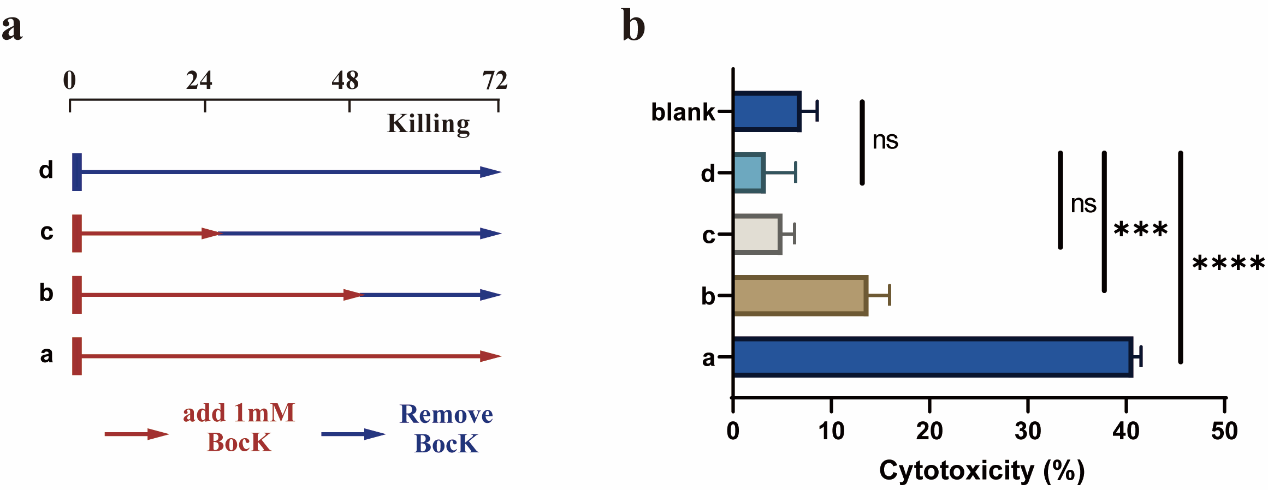


**Extended Data Figure S5. Reversible control of GCE-CAR-T cell cytotoxicity *in vitro*.**

(**a**) Schematic illustration of the BocK administration and withdrawal schedules. GCE-CAR-T cells were co-cultured with Nalm-6-fluc cells at an effector-to-target (E:T) ratio of 1:5 under different durations of 1 mM BocK exposure. (**b**) *In vitro* cytotoxicity of GCE-CAR-T cells and Mock T control cells against Nalm-6-fluc cells evaluated at 72 h. Data are presented as mean ± SD (n=3). Statistical significance was determined by one-way ANOVA for comparisons among groups a–d, and by an unpaired t-test for the comparison between group d and the blank control. ns, not significant; *** *P* < 0.001; **** *P* < 0.0001.


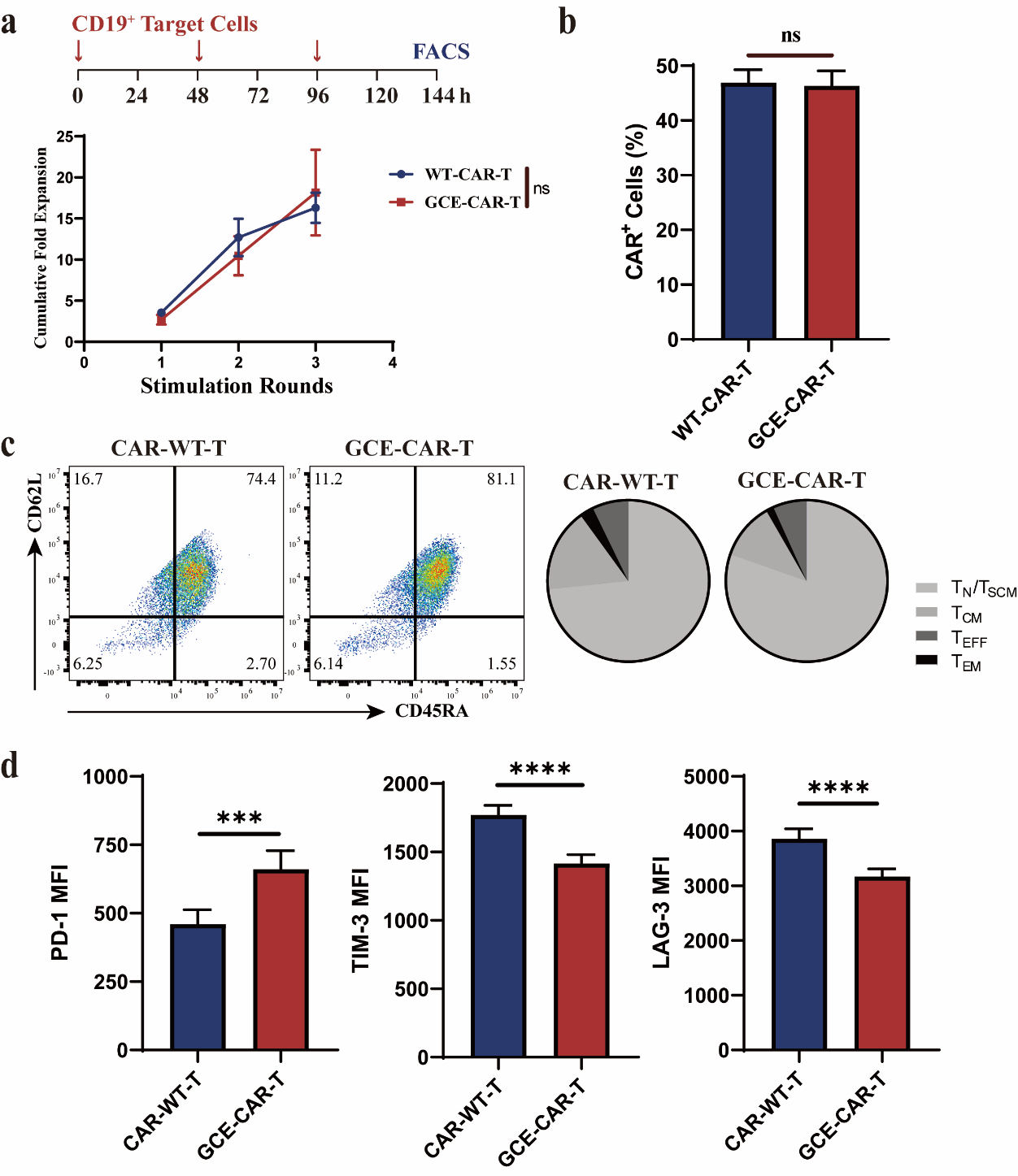


**Extended Data Figure S6. *In vitro* persistence and phenotypic characterization of CAR-T cells under chronic antigen stimulation.**

(**a**) Cumulative fold expansion of WT-CAR-T and GCE-CAR-T cells subjected to three consecutive rounds of stimulation with CD19+ target cells (red arrows indicate target cells addition). (**b**) The percentage of CAR^+^ T cells evaluated via flow cytometry at the end of the co-culture (144 h). (**c**) Representative flow cytometry plots (left) and summary pie charts (right) illustrating the distribution of T cell memory subsets based on CD45RA and CD62L expression: T_N_/T_SCM_ (CD45RA^+^CD62L^+^), T_CM_ (CD45RA^-^CD62L^+^), T_EM_ (CD45RA^-^CD62L^-^), and T_EFF_ (CD45RA^+^CD62L^-^). Data are presented as mean ±SD (n=3). Statistical significance was determined by unpaired t-test. ns, not significant.

**
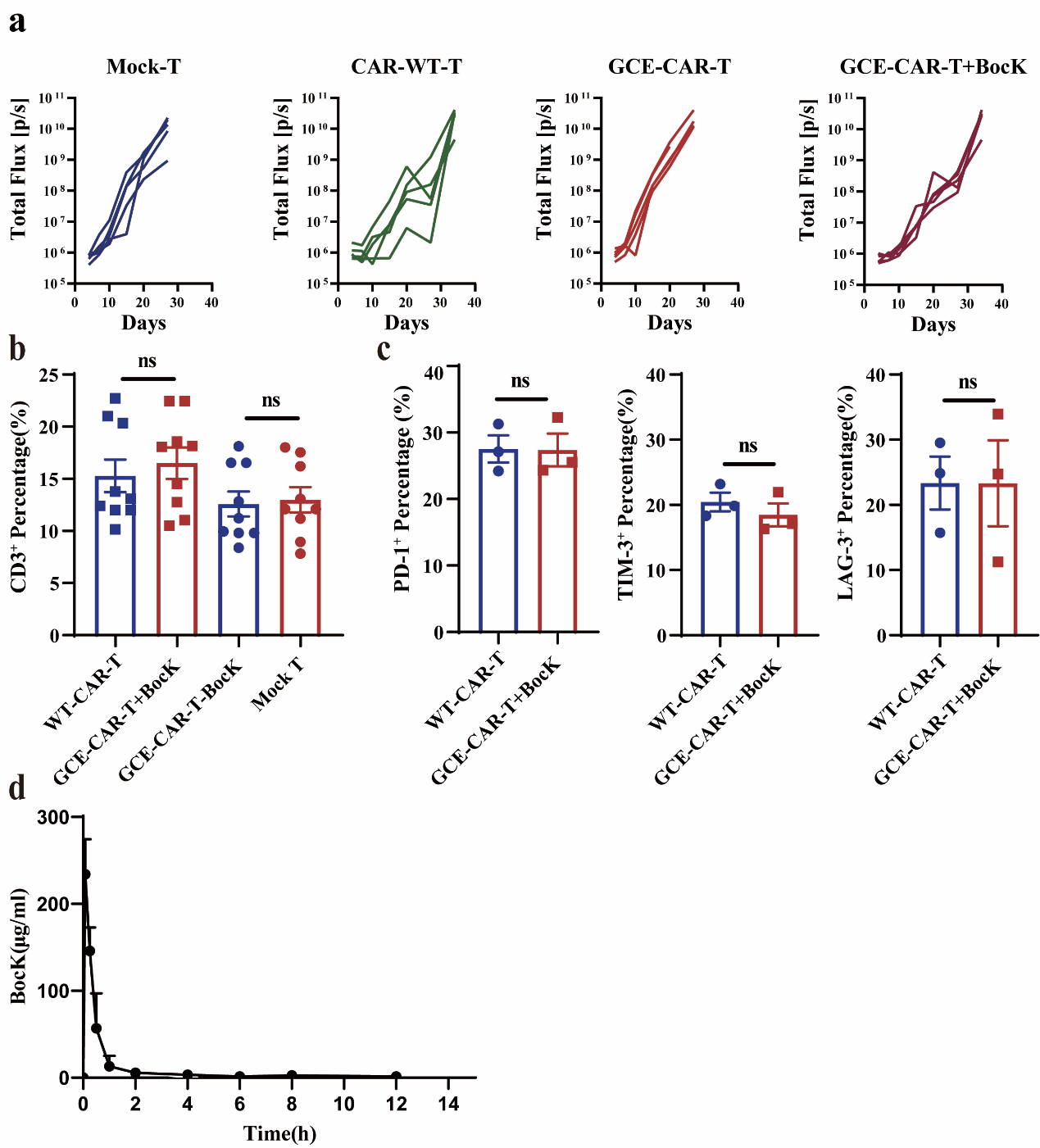
**

**Extended Data Figure S7. *In vivo* antitumor efficacy of GCE-regulated CAR-T cells in a disseminated leukemia model.**

(**a**) Total body bioluminescence flux (photons/s) for individual mice in the four experimental groups. Flux was quantified from whole-body ROIs using Living Image software. (**b**) The percentage of human CD3^+^ T cells in the spleen of Nalm-6 tumor-bearing mice at day 7 post-T cell infusion. Data are pooled from independent experiments, total n=9. (**c**) Flow cytometric quantification of the exhaustion markers on human T cells recovered *in vivo*. Data are presented as mean ±SEM (n=3 mice per group). Statistical significance was determined by one-way ANOVA for (**b**) and unpaired t-test for (**c**). ns, not significant. (**d**) *In vivo* pharmacokinetic curve of BocK from the serum following administration in mice.


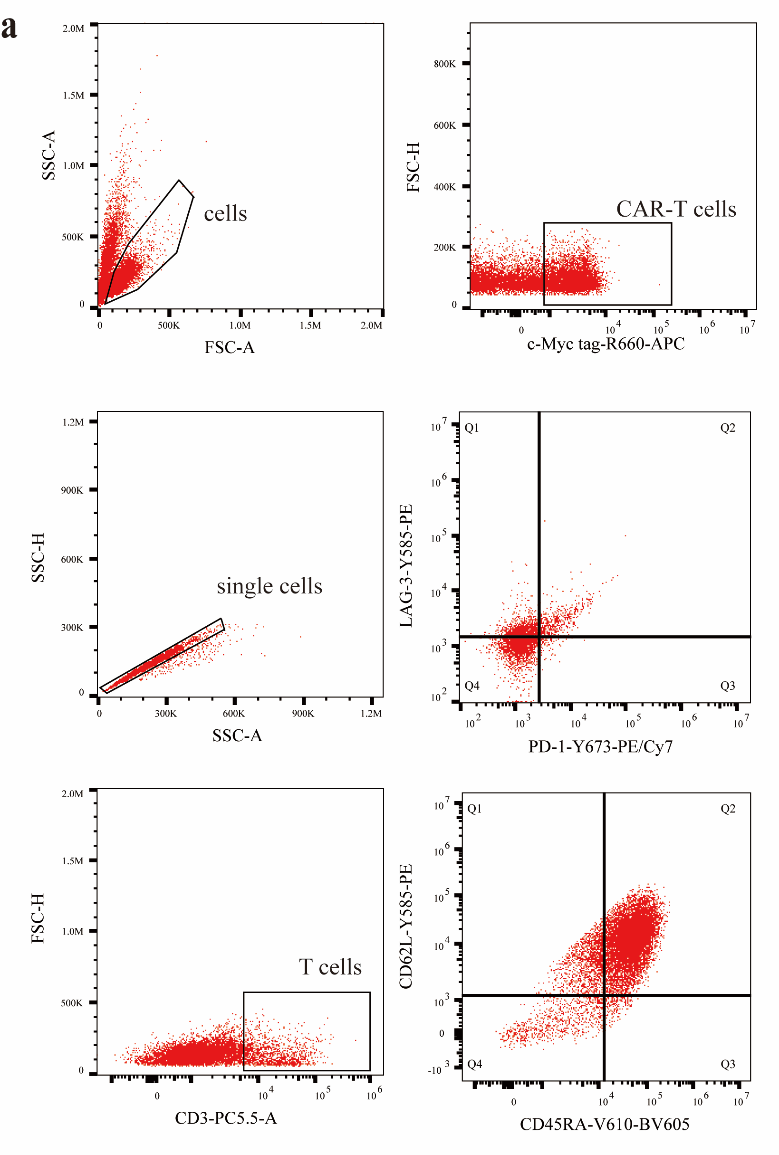


**Extended Data Figure S8. Flow cytometry gating strategy.**

(**a**) Examples of the gating strategy used for identifying and analyzing CAR-T cell populations.

**Methods**

**Plasmid construction**

The foundational wild-type (WT) anti-CD19 CAR sequence was custom-synthesized (GenScript) and inserted into a *piggyBac* transposon backbone. All CAR architectures were designed with a C-terminal EGFP fusion to facilitate subsequent expression tracking. To generate the amber codon-mutated CAR variants, overlap extension PCR was applied utilizing the WT construct as a template. The engineered tRNA sequence, PyOtR, was obtained from established literature and commercially synthesized (Genewiz)(Jewel et al., 2024). Subsequently, Gibson assembly was employed to integrate these mutant CAR genes into a modified *piggyBac* plasmid harboring tandem copies of the human U6-driven PyOtR cassette. To complete the tightly regulated GCE platform, the orthogonal synthetase (*Mb*PylRS) was independently cloned into a corresponding *piggyBac* vector that co-expresses two additional U6-PyOtR units.

**Cell culture**

Jurkat-T cells (ATCC, TIB-152), K562 cells (ATCC, CRL-3344) and Nalm-6 cells (ATCC, CRL-3273) were maintained in RPMI-1640 medium (Macgene, CM10041), supplemented with 10% fetal bovine serum (FBS; Hyclone, SV30208.02), at 37 °C and 5% CO₂ in a humidified incubator.

Jurkat-T and Nalm-6 cell lines were transfected by electroporation (Celetrix cell electroporator, CTX-1500A LE). Cells were harvested and resuspended in 20 μL electroporation buffer (Celetrix, 13-0104) at the density of 1 × 10⁶. Then, 3 μg of plasmids were mixed with the cells and electroporated under the optimized conditions (Cell line mode, 520V). After electroporation, the cells were immediately transferred to pre-warmed culture medium with or without intended BocK. Then, the cells were cultured for another 48 h and used for subsequent assays.

**Manufacturing of stable and regulated CAR-T cell lines**

Human peripheral blood mononuclear cells (PBMCs) were sourced from healthy donors by YAYU Bio, with ethical approval granted by Shanghai Liquan Hospital and written consent obtained from the donors for their blood to be used in research purposes. Untouched primary human T cells were enriched from fresh samples via negative selection utilizing the Dynabeads Untouched Human T Cells Kit (Invitrogen™, 11344D). For T cell receptor stimulation, the purified cells were co-incubated with XYbeads Human CD3/CD28 T Cell Activator (Thermo Fisher, 11131D) at a bead-to-cell ratio of 1:1. The cultures were maintained under standard incubator conditions (37 °C, 5% CO₂) in a basal medium of X-VIVO 15 (Lonza, 04-418Q). This medium was fully supplemented with 10% heat-inactivated fetal bovine serum (Gibco/Thermo Fisher, 10099141C), 50 µM β-mercaptoethanol (Macklin, M917637-10mL), and a cytokine cocktail comprising 30 IU/mL IL-2 (SinoBiological, GMP-11848-HNAE-50), alongside 5 ng/mL each of IL-7 (11821-HNAE-10ug) and IL-15 (10360-HNCE-10ug). Following a 48-hour stimulation period, the activating beads were magnetically depleted from the cell suspension.

Following beads removal, the cells were harvested and resuspended in 20 μL electroporation buffer (Celetrix, 13-0104) at a concentration of 1 × 10⁶ cells per mL. A mixture of 3 μg of plasmids (piggyBac-CAR, piggyBac-MbPylRS, and pBase plasmids at a 2.5:2.5:1 mass ratio) was added to the cells and electroporated under optimized conditions (Cell line mode, 520V). After electroporation, the cells were immediately transferred to pre-warmed culture medium, with or without BocK. The cells were cultured for an additional 48 hours, after which CAR-positive cells were sorted using the BD FACS Discover S8 flow cytometer. After a 4-day expansion period, the CAR-positive cells underwent a second round of sorting to establish a stable, regulated CAR-T cell line, which was then used for further experimental analysis.

**Flow cytometric analysis of CAR expression**

To evaluate the BocK concentration-dependent expression of CAR, 2 × 10⁵ GCE-CAR-Jurkat cells or CAR-T cells were seeded in U-bottom 96-well plates. A gradient dilution of the non-canonical amino acid BocK was added to the culture medium. To evaluate the system sensitivity and CAR expression following continuous 1 mM BocK treatment, 1 mM of BocK was added to the culture medium and removed according to the designed time. Reversible regulation of CAR expression using alternating BocK administration was evaluated by both ON-OFF-ON and OFF-ON-OFF regimens. After incubation, cells were harvested by centrifugation at 400 × g for 5 minutes. The cell pellet was resuspended in PBS. The expression of CAR was analyzed using a four-laser flow cytometer or six-laser flow cytometer (CytoFLEX, Beckman Coulter). Flow cytometry data were processed to quantify EGFP fluorescence intensities, which served as indicators of CAR expression.

**Flow cytometric analysis of Jurkat-T cell activation**

To assess the BocK concentration-dependent activation of GCE-CAR-JT cells, 2 × 10⁵ GCE-CAR-JT cells were co-cultured with K562-tCD19 or K562 cells at a 1:1 cell ratio in U-bottom 96-well plates. A gradient dilution of BocK was added to the culture. After 48 hours of co-culture, cells were collected by centrifugation at 400 × g for 5 minutes. The cell pellet was resuspended in FACS wash buffer (PBS + 2% FBS) and incubated on ice for 60 minutes to block non-specific binding. Following a second round of centrifugation, the cells were stained with an APC-conjugated anti-human CD69 antibody (Biolegend, 310910) for 30 minutes at 4°C. After staining, cells were washed three times with FACS wash buffer and subsequently resuspended in PBS. The expression of CD69 on the surface of Jurkat-T cells was analyzed using a six-laser flow cytometer (CytoFLEX, Beckman Coulter). Flow cytometry data were processed to determine the level of CD69 expression as an indicator of T cell activation.

**Coculture cytotoxicity assay**

To assess the cytotoxicity of CAR-T cells *in vitro*, a luciferase-based assay was utilized. Briefly, 1 × 10⁴ Nalm6-Luc cells were seeded into white 96-well plates (Biyuntian, FCP968). CAR-T cells were seeded alongside Nalm6-Luc target cells across predetermined effector-to-target (E: T) ratios or titrated concentrations of BocK. Following a 16-hour co-incubation period, the surviving target cell population was quantified. Specifically, the residual firefly luciferase (Fluc) luminescence was recorded utilizing a BioTek Synergy H1 microplate reader, with all experimental conditions performed in triplicate. Cytotoxicity was quantified by calculating the lysis rate using the following formula:

Cell viability (%) = [(Effector + tumor cell co-culture group) - (blank control group for background subtraction)] / [(Non-effector control group) - (blank control group for background subtraction)] × 100%.(Guldevall et al., 2016)

**ELISA analysis of cytokine release by CAR-T cells**

2 × 10⁵ CAR-T cells were co-cultured with Nalm-6-Fluc cells at a 1:1 effector-to-target ratio in U-bottom 96-well plates. A gradient dilution of the non-canonical amino acid BocK was added to the culture. After 16 hours of co-culture, cells were collected by centrifugation at 400 × g for 5 minutes, and the supernatant was collected for cytokine analysis.

Cytokine levels of IL-2 and IFN-γ were measured using commercially available human IL-2 ELISA kit (Solarbio, SEKH-0008) and IFN-γ (Solarbio, SEKH-0046) ELISA kits. Absorbance was measured using a Synergy H1 microplate reader (BioTek) to quantify the cytokine secretion levels, which reflect the activation status of CAR-T cells.(Gee, 2015)

**Chronic antigenic stress test**

CAR-T cells were co-cultured with Nalm-6-fluc tumor cells and subjected to three rounds of stimulation with fresh tumor cells every 48 hours(Eyquem et al., 2017). The cumulative fold expansion and the percentage of CAR⁺ cells were evaluated at each time point using a six-laser flow cytometer (CytoFLEX, Beckman Coulter). The T cell differentiation status was assessed via surface staining with PE-conjugated anti-human CD62L (BioLegend, 304805) and BV605-conjugated anti-human CD45RA antibodies (BioLegend, 304133) prior to analysis on a six-laser flow cytometer (CytoFLEX, Beckman Coulter).

**Xenograft mouse tumor model assay**

To assess the *in vivo* antitumor potential of regulated CAR-T cells, 6–8-week-old female NOD.Cg-Prkdc^scid^IL2rg^null^/J mice (T001475 NCG, Beijing Jicui Yaokang Biotechnology) were used. Mice were acclimated in individually ventilated cages (IVC) for 1 week to minimize transport-related stress. To establish the disseminated leukemia model, mice were intravenously engrafted with 5 × 10⁵ Nalm-6-Fluc cells suspended in 200 µL of PBS. Following a 4-day engraftment period, the animals were randomized into experimental cohorts (n =5 per group), ensuring an equivalent baseline tumor burden across all groups. Subsequently, a single dose of 5 × 10⁵ engineered T cells (in 100 µL PBS) was administered via tail-vein injection. To evaluate the *in vivo* efficacy of the translational switch, daily oral administration (gavage) of BocK (40 mg/mouse/day, prepared as 200 mg/mL in PBS) or a PBS vehicle control was initiated four hours post-T-cell transfer. Systemic disease progression was longitudinally evaluated at 7-day intervals using bioluminescence imaging (BLI) following the injection of D-luciferin (150 µL, 20 mg/mL). Subjects reaching predefined humane endpoints—such as hind limb paralysis or a body weight reduction exceeding 20%—were ethically euthanized. Quantitative BLI data, expressed as total flux (Radiance, p/s/cm²/sr), were processed and analyzed using GraphPad Prism 9.

For parallel mechanistic analysis, a satellite group (n=3 per group) was Euthanized on day 7 following CAR-T cell infusion. Splenic tissues were harvested and mechanically dissociated. Following red blood cell (RBC) lysis, lymphocytes were isolated by Mouse lymphocyte separation fluid (DAKEWE, 7211011) for detailed flow cytometric analysis. Human T-cell persistence and their functional state was assessed via surface staining with PerCP/Cyanine5.5-conjugated anti-human CD3 antibody (Biolegend, 344807), PE-conjugated anti-human CD223 antibody (Biolegend, 309305), PE-conjugated anti-human CD366 antibody (Biolegend, 345005) and PE-Cy7-conjugated anti-PD-1 antibody (Biolegend, 329917) prior to analysis on a six-laser flow cytometer (CytoFLEX, Beckman Coulter).

***In vivo* pharmacokinetic analysis**

Following BocK administration, systemic blood sampling was performed via retro-orbital bleeding at designated temporal intervals. After serum separation, sample deproteinization was achieved by introducing high-purity methanol. The mixtures were centrifuged to completely remove protein precipitates, and the resulting supernatants were diluted to ensure the analyte concentration fell within the linear dynamic range of the assay. Finally, BocK quantification was executed via LC-MS.

**Statistical analysis**

Unless otherwise mentioned, all *in vitro* data represent means ± SD of three independent biological replicates. For the animal experiments, each treatment group consisted of randomly selected mice (n=3-5), and the results are expressed as means ± SEM. Comparisons between groups were performed using two-tailed unpaired t-tests. Statistical significance was determined using GraphPad (Prism, version 9.5.0), and significance was assigned at *P < 0.05, **P < 0.01, ***P < 0.001, ****P < 0.0001. n and P values are described in the figures or figure legends. For a complete breakdown of the specific statistical models, parameter settings, and exact P value calculations utilized across different experiments, please refer to Supplementary Table S1.

**Figures**

Parts of the figures were drawn by using pictures from Biovisart (<https://biovisart.com.cn>).

**References**

1.Eyquem, J., Mansilla-Soto, J., Giavridis, T., van der Stegen, S. J. C., Hamieh, M., Cunanan, K. M., Odak, A., Gönen, M., & Sadelain, M. (2017). Targeting a CAR to the TRAC locus with CRISPR/Cas9 enhances tumour rejection. *Nature*, *543*(7643), 113-117. <https://doi.org/10.1038/nature21405>

2.Gee, A. P. (2015). Manufacturing genetically modified T cells for clinical trials. *Cancer Gene Therapy*, *22*(2), 67-71. <https://doi.org/10.1038/cgt.2014.71>

3.Guldevall, K., Brandt, L., Forslund, E., Olofsson, K., Frisk, T. W., Olofsson, P. E., Gustafsson, K., Manneberg, O., Vanherberghen, B., Brismar, H., Kärre, K., Uhlin, M., & Önfelt, B. (2016). Microchip Screening Platform for Single Cell Assessment of NK Cell Cytotoxicity. *Front Immunol*, *7*, 119. <https://doi.org/10.3389/fimmu.2016.00119>

4.Jewel, D., Kelemen, R. E., Huang, R. L., Zhu, Z., Sundaresh, B., Malley, K., Pham, Q., Loynd, C., Huang, Z., van Opijnen, T., & Chatterjee, A. (2024). Enhanced Directed Evolution in Mammalian Cells Yields a Hyperefficient Pyrrolysyl tRNA for Noncanonical Amino Acid Mutagenesis. *Angewandte Chemie International Edition*, *63*(9), e202316428. <https://doi.org/https://doi.org/10.1002/anie.202316428>
